# Clinical and Laboratory Parameters in Iraqi Alpha-Thalassemia Pediatric Patients With Different Genetic Profiles, Basrah, Iraq: A Single-Center Study

**DOI:** 10.1155/anem/5516589

**Published:** 2025-09-08

**Authors:** Rawshan Zuhair Jaber, Meaad Kadhum Hassan, Sadeq Khalaf Al-Salait

**Affiliations:** ^1^Department of Pediatrics, Center of Hereditary Blood Diseases, Basrah Health Directorate, Basrah, Iraq; ^2^Department of Pediatrics, College of Medicine, University of Basrah, Basrah, Iraq; ^3^Department of Pathology, Al-Zahraa College of Medicine, University of Basrah, Basrah, Iraq

**Keywords:** α-thalassemia, genotypes, Iraq

## Abstract

**Background:**α-Thalassemia is a type of inherited hemoglobin disorder with variable severity. Clinically, the severity varies from nearly asymptomatic to severe hemolytic anemia that is life-threatening based on the number of affected genes. Although α-thalassemia has been reported in Iraq, studies concerning phenotype–genotype correlations are lacking.

**Objectives:** Our aim was to identify the types of α-thalassemia mutations and clinical phenotypes of α-thalassemia in relation to the mutation type.

**Patients and Methods:** This analytical cross-sectional study included 84 (55 males and 29 females) patients with α-thalassemia who were ≤ 18 years old registered at the Pediatric Department–Center for Hereditary Blood Diseases, Basrah, Iraq. An analysis of α-globin defects was performed using multiplex polymerase chain reaction (PCR) and direct sequencing.

**Results:** Deletional mutations were reported in 45.24% of patients, nondeletional mutations in 3.57%, and 51.19% had both deletional/nondeletional mutations. The most frequent mutation was α_2_ poly A-1 (HbA2:c.^∗^94 A > G), which was documented for 35 (41.66%) of all mutations, followed by Mediterranean (MED) (HbA1, 2:c.−31_717del) in 29 (34.52%) patients, while the most common genotype was −^MED^/αα in 17 (20.23%) patients. Blood transfusions were required in 28 (80.00%) of those who had nondeletional HbH. Iron overload was reported in 4 (11.43%) patients with nondeletional HbH; this finding did not significantly differ from other types of alpha-thalassemia.

**Conclusions:** The most common reported mutation was α_2_ poly A-1 (HbA2:c.^∗^94A > G), followed by the MED mutation (HbA1, 2:c.−31_717del), while the most frequent genotype was −^MED^/αα. Blood transfusions were more frequent in patients with nondeletional HbH.

## 1. Introduction

Alpha-thalassemia is the most common single-gene disorder that results from over 370 different mutations of the α-globin genes [1].

The clinical phenotypes of α-thalassemia vary according to the number of affected genes from nearly asymptomatic to lethal hemolytic anemia [2]. Normally, there are two α-globin genes per haploid genome (αα/αα). The α2 gene (*HBA2*) lies upstream of the α1 gene (*HBA1*) and is expressed 2-3-fold more than the α1 gene (*HBA1*) [3]. The different expressions of these two α genes have implications for the amount of hemoglobin (Hb) variants present in carriers of α1 or α2 globin mutations and for the pathophysiology of the deletional and nondeletional forms of alpha-thalassemia [4].

Alpha-thalassemia is usually caused by deletions of α-gene clusters present on Chromosome 16. It can be divided into α^+^ thalassemia, when one α gene is deleted (−α/αα), and α^0^-thalassemia, which usually results from the deletion of two α genes (−/αα) or (−α/−α). The most common deletional form of α^+^-thalassemia worldwide is −α^3.7^, with an estimated frequency of 30%–50% [5]. HbH is a tetramer of β-chains (β4) that indicates deletion of three out of four α genes (−/−α) and can also result from structural abnormalities of the α-globin gene (−α^ND^/αα), a nondeletional form of α-thalassemia. In Hb Barts hydrops fetalis, there are no functional α genes (−/−) [2].

Heterozygotes for single α gene deletions (−α/αα) have normal clinical and hematological variables, and molecular and DNA studies are necessary for diagnosis. Individuals with α^0^-thalassemia have mild microcytic hypochromic anemia with normal Hb A_2_ levels, and these individuals usually do not require treatment [6, 7]. Patients with deletional HbH, the Hb levels range between 6.9 and 10.7 g/dL, and they do not need regular blood transfusions or iron chelation treatments, while in nondeletional patients, the Hb levels are usually lower (ranging from 3.8 to 8.7 g/dL), therefore, may require occasional or frequent blood transfusions [8, 9].

Understanding the molecular genetics and genotype/phenotype correlations in α-thalassemia is essential for building a comprehensive knowledge base, improving recognition, guiding genetic counseling, and clinical care [10]. The molecular basis of α-thalassemia in Iraq has not been widely studied, with most studies being conducted in the northern parts of the country (Kurdistan) [11–14], where the prevalence of carrier rates of α-thalassemia ranges from 0.03% to 1.22% among Iraqi Kurds [15]. There are no published data from our province or neighboring provinces in the South of Iraq. Therefore, our study aimed to identify the types of α-thalassemia among registered patients in Basrah and to look for the clinical phenotypes of α-thalassemia with respect to the mutation type.

## 2. Materials and Methods

A cross-sectional study was carried out on 84 (55 male and 29 female) children and adolescents aged 6 months–18 years who were diagnosed with α-thalassemia at the Pediatric Department–Center of Hereditary Blood Disease (CHBD) in Basrah, southern Iraq, from the first of October 2020 through March 2021.

### 2.1. Data Collection

The collected data included age at diagnosis, residence, educational level, parental consanguinity, main complaint at first presentation, reasons for screening if asymptomatic, family history of α-thalassemia, past medical and surgical history, and drug history. Complete physical examinations including anthropometry were performed on all patients.

Patients included in this study were those with early peak on capillary electrophoresis or positive HbH preparation and those with microcytic hypochromic anemia with normal iron study and Hb quantitation by capillary electrophoresis.

Patients with acute illness during the current visit and patients known to have other hemoglobinopathies, such as sickle cell disease and β-thalassemia, were excluded from the study.

The study was approved by the Ethical Committee of the College of Medicine, University of Basrah (Ref. number: 03041176-2021). Informed consent was obtained from all patients and their guardians before recruitment in the study.

### 2.2. Laboratory Methods

Two milliliters of whole blood were collected in di-potassium ethylenediaminetetraacetic acid (K2 EDTA) vacuum tubes (hematological tests and genetic tests), and 1 mL was collected in clot activator gel tubes (for biochemistry tests).

A complete blood count was performed using the CELL-DYN Ruby system, Abbott Diagnostics, USA. The Hb and red blood cell (RBC) indices, RBC count, mean corpuscular volume (MCV), mean corpuscular Hb (MCH), MCH concentration (MCHC), and red cell distribution width (RDW) were evaluated according to age and sex using reference values [16–18]. HbH preparation was performed using new methylene blue stain (as a supravital stain) by adding 2 volumes of the stain to 1 volume of blood. The mixture was incubated for 2 h and then smeared on a slide to be examined by the laboratory hematologist physician for any HbH inclusions in the RBCs [19]. For a more solid confirmation of the presence of HbH/Bart's, capillary electrophoresis was performed using a Sebia Capillary system (France).

Serological tests included liver function tests (e.g., ALT and TSB) using an Abbott Biochemistry Analyzer 4000 and serum ferritin tests using an FERR Gen.4 Cobas C kit from Roche Diagnostics (Cat. no. 4885317190). Measurements of serum ferritin were performed with a Cobas E411 Analyzer (Germany).

### 2.3. Genetic Study

For the genetic study, peripheral whole blood samples were collected from every patient in EDTA tubes and were stored at 4°C until the time of DNA extraction (within 2–5 days). For each patient, genomic DNA was extracted from 100 μL of whole blood using a ReliaPrep Blood gDNA Miniprep System kit from Promega. The results of the extraction process and the presence of DNA were confirmed using gel electrophoresis in 1% agarose gel.

For the detection of deletional mutations, the type of deletion, and/or the presence of point mutations, we followed the modified technique from Chong et al. [20] and Chow et al. [21]. Sanger sequencing was performed for the polymerase chain reaction (PCR) products that were assigned to detect point mutations in the α1 and α2 genes, and mutations were then detected by aligning and comparing the query sequences with the reference sequences of the α1 and α2 genes using the NCBI website.

The studied patients were classified according to the clinical types of α-thalassemia according to the Thalassemia International Federation (TIF) guidelines [1].

### 2.4. Statistical Analysis

All statistical analyses were carried out using the Statistical Packages for Social Sciences (IBM SPSS Corp., Armonk, NY.) software Version 20.0. Categorical variables are presented as numbers and percentages, while scale variables are presented as means and standard deviations (SDs) or standard errors (SEs). Comparisons of proportions were performed by crosstab using the chi-square test and Fisher's exact test. For comparisons of the means of selected hematological and biochemical parameters in relation to the different mutation types of alpha-thalassemia, one-way analysis of variance (ANOVA) was used.

## 3. Results

A total of 84 children and adolescents were enrolled in this study, and their ages ranged from 6 months to 18 years, with a mean age of 8.71 ± 3.62 years and a male-to-female ratio of 1.89. Most patients (67.86%) were born to parents who were relatives, Table 1.

Compound heterozygous was the most commonly reported α-thalassemia genotype (51.19%), followed by deletional genotype (45.24%), and the least common was the nondeletional type, Table 2. The most common deletional mutation was Mediterranean (MED) (HbA1, 2:c.−31_717del) 29 (34.52%), followed by −(α)^20.5^ (28.57%) and −α^3.7^ (19.05%). Alpha_2_ poly A (HbA2:c.^∗^94A > G) was the most frequent nondeletional mutation type (41.66%).

The most frequent genotypes were −^MED^/αα in 20.23% of individuals, −(α)^20.5^/αα^poly A^ in 19.06% of individuals, and both (−^3.7^α/αα) and (−(α)^20.5^/αα) in 7.15% of individuals, Table 2.

Most patients were symptomatic at the time of diagnosis (91.67%), and the most common complaint was pallor (71.43%). Most of these symptomatic patients had nondeletional HbH. Asymptomatic patients who visited the CHBD for evaluation (8.33%) were mainly assessed because they had other family members with α-thalassemia.

Previous hospitalizations were reported in 49 (58.33%) patients. The most common cause of hospitalization was anemia in 32 (38.09%) patients, followed by infections in 17 (20.23%) patients. The frequencies of anemia and blood transfusions were significantly higher among those with nondeletional HbH, while infections were more common among those with deletional HbH(*p* < 0.05, Table 3).

The means of MCV, MCH, and absolute reticulocyte count were found to be significantly higher among patients with nondeletional alpha-thalassemia carriers, while patients with nondeletional HbH had the highest HbH percentage and the lowest HbA levels, *p* < 0.05, Table 4.

The mean Hb level, RBC count, and HbA were lower, while the mean ferritin was significantly higher in homozygous alpha^+^ thalassemia genotypes compared to other genotypes(*p* < 0.05). Patients with a compound heterozygous genotype had the highest MCV and HbH(*p* < 0.05, Table 5).

## 4. Discussion

This study assessed the genotype–phenotype correlations in α-thalassemia patients in Basrah, southern Iraq. Patients with compound heterozygous genotype constituted 51.19% of the studied α-thalassemia patients, followed by deletional genotype (45.24%) and nondeletional genotype (3.57%). The study also revealed that MED (HbA1, 2:c .31_717del) was the most common deletional mutation, followed by −(α)^20.5^ (HbA1, 2:c.−1000_−871del130) and −α^3.7^ (HbA1 or 2:deletion [3700pb]), while α_2_ poly A (HbA2:c.^∗^94A > G) was the most frequent nondeletional mutation.

The overall rate of consanguineous marriage reported by Tadmouri et al. in Iraq ranges from 47% to 60% [22], and this is comparable to the frequency of parental consanguinity in our study, although another study in Iraq reported a higher frequency (78.8%) in patients with different types of thalassemia [23]. This high rate of consanguineous marriage is one of the contributing factors for the high rate of thalassemia in Iraq.

The types of α-thalassemia mutations differ in different geographic areas. Ten types of mutations were reported in this study, including deletional mutations as single gene deletions in −α^3.7^ (HbA1 or 2:deletion [3700pb]) and −α^4.2^ (HbA1 or 2:c.−207_−1840del) and double gene deletions in MED (HbA1, 2:c.− 31_717del), −(*α*)^20.5^ (HbA1, 2:c.−1000_−871del130), SEA (HbA1:c.−27_717del), and THAI (NC_000016.10:g.149863_183312del) mutations, while the nondeletional mutations detected were α_1_cd^59^ (HbA1:c.179G > A), α_2_^polyA^ (HbA2:c.^∗^94A > G), α_2_^IVS1(−5nt)^ (HbA2:c.95 + 2 − 95 + 6del), and α2cd^142^ (HbA2:c.427T > C) (Hb CS). Most reported α-thalassemia mutations are deleterious, with many of these mutations resulting from deletions at different lengths of the alpha-globin locus. Nondeletional mutations are less common, and more than 70 forms, including point mutations, have been reported to cause deficient gene expression [24, 25].

In general, the α_2_ poly A-1 (HbA2:c.^∗^94A > G) mutation was the most frequent mutation, and it was present either in combination with other deletional mutations or as a nondeletional form. In northern Iraq, Shamoon et al. reported that the MED and −α^3.7^ deletions were the two most frequently encountered mutations, while the α2 poly A (HbA2:c.^∗^94A > G) mutation was reported as the third most common mutation and was found in 17% of cases [13]. However, our results are similar to those reported in Kuwait by Adekile et al., who reported poly A-1 mutation in 65.6% either in homozygous or heterozygous forms [26]; this may reflect the social interaction between families of the two countries and/similar ethnicity. In Saudi Arabia, the most frequent abnormality of α-globin genes was −α^3.7^ (62.3%), followed by α2^IVS1(−5nt)^ (20.7%) and α2 polyA-1 (α2^T.Saudi^) (14.1%) [27]. Our results differ from those seen in Northeast Peninsular Malaysia, where the deletional mutations were the most common (57.8%), followed by nondeletional (28.1%) and compound heterozygous (14.1%) [24]. In Thailand, the most common mutation was SEA (43%), while the nondeletional Hb constant spring accounts for 4.6% of cases [28].

Clinical features such as anemia and blood transfusions were more common in the nondeletional type HbH. This result differs from the results of Shamoon et al. obtained in northern Iraq, where both deletional and nondeletional types had comparable frequencies of blood transfusion (48.4% and 46.2%, respectively) [13]. Nondeletional disease is more often associated with a more severe phenotype, transfusion dependence, and the need for regular iron chelation compared to the deletional type [10, 26]. This can be attributed to the greater globin chain imbalance in nondeletional mutations than for the deletional types and because nondeletional mutations give rise to unstable α-globin chains, resulting in more severe anemia [29].

Although the Hb levels were low in all types of studied α-thalassemia patients, those with the deletional genotype (carrier or HbH) had higher Hb A levels and significantly lower MCV and MCH levels than those with the nondeletional genotype. Different studies have reported conflicting results. Pornprasert et al. reported that patients with deletional HbH disease had higher RBC counts, total Hb, MCH, MCHC, HbA, and HbA2 levels than those with nondeletional HbH disease [30]. These results are in contrast to those reported by Bozdogan et al. in southern Turkey, which did not reveal significant differences between deletional and nondeletional mutations (*p* > 0.05) [31]. Hashemi-Soteh et al. reported lower mean MCV values for deletional mutations (67.4–77.7 fL) compared to nondeletional mutations (69.2–85.7 fL) in Northern Iran [32]. In general, deletional mutations cause relatively mild anemia, with prominent microcytosis and hypochromia, while the nondeletional forms result in moderate to severe disease characterized by ineffective erythropoiesis, recurrent transfusions, and growth retardation [2].

Serum ferritin levels were not significantly different between higher α-thalassemia carriers and HbH, whether deletional or nondeletional, a result similar to that reported by Shamoon et al. in Northern Iraq [13].

On classifying patients into those with heterozygous α^0^-thalassemia, heterozygous deletional α^+^-thalassemia, homozygous deletional α^+^-thalassemia, and compound heterozygotes (deletional/nondeletional), we observed that alpha + homozygous genotype had the lowest Hb and RBCs and highest mean of ferritin; this can be explained by that poly A homozygous genotype is more severe and may behave as HbH, a result similar to that reported in United Arab Emirates [33].

One of the limitations in this study is the small number of patients assessed to represent the magnitude of the problem in Basrah and the need for extended genetic laboratory investigations to address different types of mutations.

In conclusion, the most commonly reported mutation was α2 poly A-1(HbA2:c.^∗^94A > G), followed by the MED mutation, while the most frequent genotype was −^ME^D/αα. Blood transfusions were more frequent in patients with a compound heterozygous genotype, followed by a nondeletional genotype, where iron overload occurred more frequently than for the other genotypes.

## Figures and Tables

**Table 1:** Sociodemographic characteristics of patients with α-thalassemia.

Variables			Number (*N*) Total *N* (84)	%
Age (Y)	Less than 5		16	19.05
	5–10		36	42.86
	> 10–15		30	35.71
	> 15–18		2	2.38
	Mean ± SD		8.71 ± 3.62	
	Minimum–maximum		1.67–15.75	
	IQR		4.83	

Sex	Male		55	65.48
	Female		29	34.52

Residence	Periphery		26	30.95
	Center		58	69.05

Educational level	Preschool		19	22.62
	Primary		52	61.90
	Secondary		12	14.29
	Illiterate		1	1.19

Parental consanguinity	Relatives	1^st^ cousin	37	44.05
		Beyond 1^st^ cousin	20	23.81
	Not relatives		27	32.14

Abbreviation: IQR, interquartile range.

**Table 2:** Frequencies of different α-thalassemia genotypes.

Genotype	Number (*N*. 84)	%	Confidence interval (95%)	
			Lower	Upper
*Deletional*				
−^MED^/αα	17	20.23	11.9	28.6
−α^3.7^/αα	6	7.15	2.4	13.1
−(α)^20.5^/αα	6	7.15	2.4	13.1
−^SEA^/αα	4	4.76	1.2	9.5
−^MED^/−α^4.2^	2	2.38	0	6.0
−^THAI^/αα	1	1.19	0	3.6
−α^3.7^/−α^3.7^	1	1.19	0	3.6
−α^3.7^/−^SEA^	1	1.19	0	3.6
Total	38	45.24	33.3	56.0

*Nondeletional*				
αα^polyA−1^/αα	1	1.19	0	3.6
αα^polyA−1^/αα^polyA−1^	1	1.19	0	3.6
α^cd59^*α*/αα	1	1.19	0	4.7
Total	3	3.57	0	8.3

*Compound heterozygotes*				
−(*α*)^20.5^/αα^polyA−1^	16	19.06	10.7	27.4
−^MED^/αα^polyA−1^	5	5.95	1.2	11.9
−α^3.7^/αα^polyA−1^	5	5.95	1.2	11.9
−^SEA^/αα^polyA−1^	5	5.95	1.2	11.9
−(*α*)^20.5^/αα^IVS1(−5nt)^	2	2.38	0	6.0
−^MED^/αα^IVS1(−5nt)^	2	2.38	0	6.0
α^cd59^*α*/−^MED^	2	2.38	0	6.0
−^THAI^/αα^polyA−1^	2	2.38	0	6.0
−α^3.7^/αα^cd142^	2	2.38	0	6.0
−^MED^/αα^cd142^	1	1.19	0	3.6
−α^3.7^/αα^IVS1(−5nt)^	1	1.19	0	3.6
Total	43	51.19	40.5	61.9

**Table 3:** Clinical differences in selected clinical data among genotype groups.

Variable		α-Thalassemia carriers (*N*. 46)		HbH (*N*. 38)		*p* value^∗^
		Deletional (*N*. 35) *N*. (%)	Nondeletional (*N*. 11) *N*. (%)	Deletional (*N*. 3) *N*. (%)	Nondeletional (*N*. 35) *N*. (%)	
Complaint at diagnosis	Symptomatic	29 (82.86)	11 (100.00)	3 (100.00)	34 (97.14)	0.135
	Asymptomatic	6 (17.14)	0 (0.00)	0 (0.00)	1 (2.86)	

Age at diagnosis (Y)	Less than 5	25 (71.43)	6 (54.55)	2 (66.67)	26 (74.29)	0.399
	5–10	7 (20.00)	4 (36.36)	1 (33.33)	9 (25.71)	
	> 10–15	3 (8.57)	1 (9.09)	0 (0.00)	0 (0.00)	

Blood transfusion		7 (20.00)	7 (63.64)	1 (33.33)	28 (80.00)	< 0.001

Iron overload		1 (2.86)	2 (18.18)	0 (0.00)	4 (11.43)	0.181

Splenomegaly		5 (14.28)	3 (27.27)	0 (0.00)	8 (22.86)	0.684

Previous hospitalization	Anemia	7 (20.00)	6 (54.55)	0 (0.00)	19 (54.29)	0.012
	Infections	7 (20.00)	2 (18.18)	2 (66.67)	6 (17.14)	

Positive HbH preparation		33 (94.28)	11 (100.00)	3 (100.00)	33 (94.28)	1.000

BMIZ	Normal	32 (91.43)	8 (72.73)	1 (33.33)	32 (91.43)	0.015
	Thinness	0 (0.00)	0 (0.00)	0 (0.00)	0 (0.00)	
	Severe thinness	0 (0.00)	0 (0.00)	0 (0.00)	1 (2.86)	
	Over weight	3 (8.57)	3 (27.27)	1 (33.33)	2 (5.71)	
	Obese	0 (0.00)	0 (0.00)	1 (33.33)	0 (0.00)	

Height for age	< 3^rd^ percentile	3 (8.57)	3 (27.27)	0 (0.00)	8 (22.86)	0.107
	3^rd^–97^th^ percentile	32 (91.43)	7 (63.64)	3 (100.00)	27 (77.14)	
	> 97^th^ percentile	0 (0.00)	1 (9.09)	0 (0.00)	0 (0.00)	

Abbreviation: BMIZ, body mass index Z score.

^∗^ *p* value assessed by Fisher's exact test.

**Table 4:** Selected hematological and biochemical parameters in studied individuals with various α-thalassemia types.

Variables	α-Thalassemia carriers (*N*. 46)		HbH (*N*. 38)		*p* value
	Deletional (*N*. 35) Mean ± SD	Nondeletional (*N*. 11) Mean ± SD	Deletional (*N*. 3) Mean ± SD	Nondeletional (*N*. 35) Mean ± SD	
Hb (g/dL)	8.13 ± 1.40	7.46 ± 1.68	7.71 ± 1.49	8.23 ± 1.23	0.411
RBC (^∗^10^12^/L)	5.04 ± 0.86	4.21 ± 0.96	4.84 ± 0.87	4.80 ± 0.80	0.600
MCV (fL.)	54.89 ± 5.87	61.19 ± 6.82	55.63 ± 3.03	60.11 ± 7.96	0.008
MCH (pg.)	15.79 ± 2.10	17.77 ± 3.12	15.90 ± 1.32	17.38 ± 2.68	0.030
MCHC (g/dL)	28.38 ± 3.05	28.96 ± 3.15	28.66 ± 2.73	28.96 ± 2.50	0.845
RDW-CV (%)	22.04 ± 4.44	22.69 ± 5.50	24.50 ± 4.66	23.78 ± 4.73	0.473
Absolute retic. count (^∗^10^9^/L)	235.51 ± 21.78^∗^	593.73 ± 246.66^∗^	272.80 ± 35.49^∗^	276.08 ± 15.05^∗^	0.011
Hb A (%)	89.45 ± 8.06	87.27 ± 7.11	91.56 ± 4.53	82.75 ± 12.99	0.048
Hb A_2_ (%)	1.22 ± 0.46	1.20 ± 0.61	1.26 ± 0.15	1.28 ± 0.72	0.995
Hb H	6.33 ± 0.91^∗^	10.44 ± 2.75^∗^	4.90 ± 2.80^∗^	15.21 ± 2.33^∗^	0.003
Hb Barts	2.20 ± 0.53^∗^	2.00 ± 0.33^∗^	4.20	2.26 ± 0.43^∗^	0.745
Hb F (%)	1.77 ± 0.40^∗^	4.15 ± 3.25^∗^	2.60	0.97 ± 0.40^∗^	0.156
S. Ferritin (ng/mL)	159.19 ± 68.66^∗^	384.49 ± 210.13^∗^	179.83 ± 72.91^∗^	319.11 ± 87.89^∗^	0.444
TSB (mg/dL)	1.49 ± 0.25^∗^	1.30 ± 0.94^∗^	1.09 ± 0.45^∗^	1.92 ± 0.24^∗^	0.368
ALT (U/L)	18.94 ± 8.39	14.64 ± 4.99	22.33 ± 11.06	17.28 ± 10.30	0.432

Abbreviations: ALT, alanine aminotransferase; Hb, hemoglobin; MCH, mean corpuscular hemoglobin; MCHC: mean corpuscular hemoglobin concentration; MCV, mean corpuscular volume; RBC, red blood cell; RDW, red cell distribution width; SE, standard error; TSB, total serum bilirubin.

^∗^ *p* value was assessed by the ANOVA test.

**Table 5:** Selected hematological and biochemical parameters among α-thalassemia genotypes.

Variables	Alpha^+^ heterozygous (*N*. 8) Mean ± SD	Alpha^+^ homozygous (*N*. 2) Mean ± SD	Heterozygous alpha^0^ (*N*. 28) Mean ± SD	Compound heterozygous (*N*. 46) Mean ± SD	*p* value
Hb (g/dL)	7.40 ± 1.18	5.63 ± 3.98	8.27 ± 1.43	8.15 ± 1.14	0.030
RBC (^∗^10^12^/L)	4.59 ± 1.08	3.40 ± 1.54	5.09 ± 0.88	4.75 ± 0.76	0.032
MCV (fL.)	54.82 ± 9.09	58.20 ± 4.38	55.32 ± 6.07	60.01 ± 7.34	0.033
MCH (pg.)	16.54 ± 3.08	15.50 ± 4.66	15.81 ± 2.17	17.37 ± 2.57	0.073
MCHC (g/dL)	30.11 ± 1.73	26.40 ± 5.93	28.10 ± 3.25	28.97 ± 2.43	0.182
RDW-CV (%)	21.71 ± 3.03	29.15 ± 3.88	21.89 ± 4.67	23.52 ± 4.79	0.105
Absolute retic. count (^∗^10^9^/L)	156.05 ± 41.73^∗^	266.27 ± 68.67^∗^	254.81 ± 23.95	353.93 ± 61.25^∗^	0.333
Hb A (%)	83.61 ± 10.58	83.50 ± 1.55	91.52 ± 1.55	83.98 ± 12.00	0.020
Hb A_2_ (%)	1.57 ± 0.25^∗^	1.60 ± 0.60^∗^	1.15 ± 0.06^∗^	1.21 ± 0.09^∗^	0.266
Hb H	9.74 ± 4.33^∗^	10.15 ± 5.75^∗^	5.09 ± 0.62^∗^	13.96 ± 1.86^∗^	0.004
Hb Barts	2.20 ± 0.35^∗^	0.70	2.33 ± 0.60	2.25 ± 0.34^∗^	0.896
Hb F (%)	3.03 ± 2.18^∗^	0.00	1.93 ± 0.43^∗^	1.17 ± 0.40^∗^	0.316
S. Ferritin (ng/mL)	474.99 ± 296.19^∗^	1345.15 ± 1015.85^∗^	84.72 ± 17.77^∗^	274.94 ± 67.92^∗^	0.001
TSB (mg/dL)	0.75 ± 0.07^∗^	0.75 ± 0.15^∗^	1.66 ± 0.31^∗^	1.83 ± 0.20^∗^	0.192
ALT (U/L)	17.51 ± 5.88	22.50 ± 3.53	19.25 ± 8.91	16.78 ± 9.63	0.608

Abbreviations: ALT, alanine aminotransferase; Hb, hemoglobin; MCH, mean corpuscular hemoglobin; MCHC, mean corpuscular hemoglobin concentration; MCV, mean corpuscular volume; RBC, red blood cell; RDW, red cell distribution width; SE, standard error; TSB, total serum bilirubin.

^∗^ *p* value was assessed by the ANOVA test.

## Data Availability

The data that support the findings of this study are available from the corresponding author upon reasonable request.
